# Supplementary material for: Effect of influenza vaccination on resting metabolic rate and c-reactive protein concentrations in healthy young adults
Source: PLoS One. 2023 Dec 15;18(12):e0295540. doi: 10.1371/journal.pone.0295540 (PMC10723728; doi:10.1371/journal.pone.0295540)
Supplement: Attachment — Submitted filename: Response to Reviewers.docx [file pone.0295540.s002.docx]

**PONE-D-23-11732**

**Response to Reviewers**

Dear Dr. Loor,

Thank you for giving us the opportunity to submit a revised draft of the manuscript “Effect of influenza vaccination on resting metabolic rate and c-reactive protein concentrations in healthy young adults” for publication in PLOS ONE. We are grateful to both you and the reviewer for the feedback you have provided, which has substantially improved our paper. We removed the funding-related text from the Acknowledgments section of our manuscript since it had already been included in the Funding Statement. As a result, the Funding Statement does not need to be updated. We have addressed and incorporated all the suggestions made by the reviewer. Those changes are marked-up in the manuscript. Please also see below for a point-by-point response. All line numbers refer to the revised manuscript file with tracked changes.

**Response to Reviews**

**Reviewer 1**

The study was carried out in a comprehensive cohort, investigation methods are chosen wisely and using R for statistical analysis is appealing. Even following an as mild stimulation as an influenza vaccination the authors detected elevation in CRP level while the resting metabolic rate did not change.

**Author response**: Thank you!

1. All the participants of the study received influenza vaccination in earlier years. It would have been interesting to include participants without any such experience. Other kind of vaccination patients could have been included as well to gain a more nuanced picture, e.g. patients receiving tetanus vaccination, tick encephalitis vaccination or others.

**Author response**: We agree that this is a limitation of our study. We have included an additional sentence in the Conclusions and future directions section to suggest future studies evaluate multiple types of vaccines.

The revised text reads as follows on lines 317-321:

“Further, future studies should evaluate the immune response in vaccine naïve patients (i.e., not previously exposed to a given vaccination). Other vaccinations beyond influenza (e.g. tetanus, tick encephalitis, COVID-19, etc.) may elicit different or stronger immune responses and should also be considered when designing future studies.”

2. As the authors also pointed out in the discussion, RMR was reported to peak at six to eight hours after vaccination but no measurements were taken at that time.

**Author response**: We agree that this is a limitation of our study. However, we believe we have sufficiently addressed it in our discussion and conclusions and future directions sections. Our study design aligned with previous studies evaluating the effect of influenza vaccination on CRP, where peak CRP is expected two days post-vaccination. The only studies of vaccination effects on RMR have been conducted with the typhoid vaccine, which differs in several ways from the influenza vaccine. Therefore, we choose to follow the existing influenza vaccination literature when designing this study. It may be worth developing future studies to measure RMR much earlier after injection.

3. While in the text mean CRP levels are mentioned, Figure 1 shows the median CRP level instead. CRP values are not normally distributed after the stimulus.

**Author response**: We agree with the reviewer’s assessment. Accordingly, throughout the manuscript, we have reported both mean ± SD and median (IQR) CRP and RMR levels to account for the non-normal distribution. Additionally, we included the following text on lines 170-171:

“Day two and day seven CRP levels were right-skewed, with a few participants exhibiting elevated CRP far above the IQR (Fig. 1).”

4. Figure 3 is not informative. The authors should discuss in further details what the result on the plot shows and how does it affect the drawn consequences. Did the authors test if and how the changes in the ratio of CRP levels correlate with the changes in the ratio of RMR? Showing the differences on the axes of the plot can be misleading since these values are dependent on the scale of the measurement.

**Author response**: We agree with the reviewer’s assessment that Figure 3 did not add valuable information to our paper and have decided to remove it from the manuscript.

5. In Figure 4, patients are divided based on CRP level. Elevated CRP level was defined as CRP>3.0 mg/ml. Could the authors rationalize this choice? Is this CRP level at day 0 (before vaccination)? On the plot are the day 2/ day 0 and day 7/ day 0 ratios combined?

**Author response:** As suggested by the reviewer, we clarified our reasoning for defining the elevated CRP level as CRP>3.0 mg/ml. The revised text reads as follows on lines 276-278:

“Individuals with CRP levels between 3.0 mg/L and 10.0 mg/L are considered to have low-grade inflammation [15,22,27]. As such, we considered participants with CRP>3.0mg/L as having elevated CRP”

We also updated the language in the description of the figure (now Figure 3) to clarify how variables were used for analysis. The revised text reads as follows on lines 287-289:

“RMR was measured at baseline (before vaccination) and at days two and seven after vaccination; the day two:baseline and day seven:baseline ratios were combined for analysis. CRP measurements on days two and seven after vaccination were combined and subdivided as Elevated (CRP>3.0 mg/L) or Low (CRP≤3.0 mg/L).”

6. In line 190, does BGL refer to blood glucose level? Please open the abbreviation.

**Author response:** We opened the abbreviation of BGL to blood glucose level (line 196).

7. It is misleading to read about a „1.0-fold increase” (line 265) or „an average increase in CRP of 1.47 mg/L, representing a 106% elevation from baseline” (line 212).

**Author response:** We replaced references to fold increases with percentage increases in the introduction (line 63) and the discussion (line 272).
